# Medical Treatment Prior to, or in Connection with, Filling Teeth

**Published:** 1888-02

**Authors:** G. E. Corbin

**Affiliations:** St. Johns, Mich.


### What Medical Treatment Is Frequently Required Prior to, or in Connection with, Filling Teeth?

BY G. E. CORBIN, M.D., D.D.S., ST. JOHNS, MICH.

From a careful consideration of the phraseology of this question, I entertain the opinion that it may be answered without attempting to enumerate *all* of the conditions which require medical treatment prior to the filling of teeth.

Soft and fragile teeth of invalids frequently become more dense and stronger as the general bodily health improves under the influence of proper constitutional treatment.

Whether such a change really takes place in the *enamel* of fully developed teeth is yet a question in dispute; but *dentine* certainly sustains such relations to the nutrient vessels of the pulp as to be improved or deteriorated by the character of the circulating medium.

For these reasons it not unfrequently happens that a long course of medical treatment, both eliminating and supporting, becomes very essential, and very beneficial, prior to any thorough attempt at permanent preservation of mouths full of badly diseased teeth. From long custom and tacit general consent, this treatment is usually left to the province of some practitioner of general medicine.

With one exception this is all right, and, doubtless for the best. With scarcely an exception, physicians, simply as such, administer remedial agents with reference to their ultimate effects on the diseases prescribed for, and without the least thought of, or regard for their chemical or other local effects on the teeth.

Muriated tincture of iron, Horsford's acid phosphates, and the like, are exceedingly destructive to the enamel of teeth, and yet physicians frequently prescribe them when other remedies, not in the least injurious to the teeth, would fulfill the general indications just as well.

The dentist's duty in reference to such teeth as are now under consideration, consists, *primarily* in warning his patient against the destructive effects on the teeth, of the mineral acids generally, and the stronger vegetable acids, both free and combined, as they may be, or may have been prescribed for him. Having emphasized this matter, he should thoroughly cleanse the teeth, remove superficial abrasions, carefully fill all cavities with some of the plastics, and await the results of proper medication before attempting elaborate gold fillings.

In various other conditions, if successful, the medical treatment certainly must be conducted by the dentist.

For example, the teeth in a bad case of alveolar pyorrhœa, falling to the care of the average practitioner of general medicine, would most certainly be extracted. Not understanding the exact pathological condition, his application of astringents "to harden the gums," so frequently made, would be of no avail, and extraction would follow. *No one* can perform miracles. The teeth in this affection when first seen by the dentist are often so extremely loosened as to make extraction the only alternative; nevertheless, in a large majority of cases successful treatment is possible. Notwithstanding this disease when fully established, with pus oozing from the margins of the gums at necks of each and every tooth in the mouth is loathsome beyond description; its treatment is simple and efficient, and consists, in the main, like that of all other diseases, simply in *removing its cause.* There is *always* a local or exciting cause.

Sometimes, but *not* always, there is a predisposing cause.

The predisposing cause would be constitutional and naturally fall to the care of the general practitioner.

The *dentist* must assume the responsibility of the *local* treatment, which is much more frequently demanded, and of paramount importance. I do not dispute that in some exceptional cases it may be *possible*, but I have yet to be convinced that any kind or process of treatment will, with any sort of certainty or uniformity, cause wasted alveolar process to be reproduced.

Where the teeth have not been loosened beyond the possibility of being reclaimed, I have found satisfactory success in the following simple treatment:

*First*, thoroughly remove all tartar, salivary calculus, sanguinary calculus—every particle of foreign substance of whatever nature, from the necks of the teeth to the very bottom of the deep pockets formed by separation of the gums. Where the gums are greatly hypertrophied, as well as separated from the teeth, the tumefied projecting festoons may be snipped off all around with sharp pointed scissors. The bleeding thus caused will reduce both their volume and their inflammation. The congested capillary blood vessels being thus so generally severed, hyperœmia and the process of hypertrophy are at once and completely arrested.

The bleeding thus induced should be encouraged for a short time. While waiting for this, the teeth, if much loosened, should be wired or tied securely in place. All coagulated blood should then be removed by syringing with tepid water, or, what is perhaps better, a weak solution of chloride of sodium.

With a small pledget of cotton on the point of a delicate and suitable instrument I carefully yet thoroughly smear all pus-producing surfaces with the old and reliable article, creosote. I

am well aware that peroxide of hydrogen and some other new remedies are more popular just at the present time, but creosote carefully and moderately applied will fulfill all the indications in this and in some other cases, and do it most perfectly. It should be applied undiluted but not in excessive quantity. It effectually destroys a thin film of diseased surface, and *never* acts deeply. It coagulates the albumen of the surface tissues, forming a thin pellicle which remains for several hours as a protection to the sensitive tissues beneath, and when thrown off reveals a healthful granulating surface in nearly all cases, if bodily condition be good.

Having secured such a condition of the parts, these healthful granulations must, so far as practicable, be protected from irritating secretions, and especially from solid particles of food. This can be well accomplished by loosely and smoothly packing the spaces between the gums and necks of teeth with cotton which has been soaked in some antiseptic preparation. I have generally used phenol-sodique considerably diluted, and find that it answers an excellent purpose. This cotton should be daily removed, the spaces syringed and again filled as before; being careful to use no more cotton than is actually required to exclude the food. It may or may not be necessary to make a second application of the creosote. Daily observation of the condition found will determine that question.

As irritation from the pressure and friction of foreign substances accumulated on the necks and roots of the teeth is the principle cause of this difficulty; so, perfect cleanliness—complete exclusion of foreign snbstances, is the one essential and indispensable feature in its successful treatment.

By following the simple course of treatment here briefly outlined, I have been astonished at the rapidity and extent of recovery.

It is exceedingly rare to see an alveolar abscess adjoining the roots of teeth with living pulps.

Aside from occasional mechanical injuries, almost invariably the death and decomposition of the pulp of a tooth is the cause of alveolar abscess; and the *treatment* consists in the removal of the cause; therefore the sooner the pulp chamber and root canals can be thoroughly cleansed and filled, the better.

If the abscess be of long standing and drained through a fistulous opening, the sinus should be probed. If carious, or necrosed bone be detected, it should be removed. The cavity should then be cleansed by syringing, and the pus producing surfaces destroyed by creasote or iodine, which will cause granulation and healing from the bottom. The practice of treating alveolar abscess through the roots of teeth is sometimes successful, but many times actually detrimental.

Medicated pledgets of cotton thrust far up in the roots, in my opinion, have done much more harm than good, for they prevent the escape of pus and thereby prolong and aggravate the difficulty.

Syringing through the roots, to apply antiseptics and to aid in the discharge of pus is, many times, the best of treatment; but as soon after this has been accomplished, as practicable, the roots should be permanently *cleansed* and *closed*.

Should any further treatment be necessary, it can best be made through the thin alveolus.

